# Research on machine learning forecasting and early warning model for rainfall-induced landslides in Yunnan province

**DOI:** 10.1038/s41598-024-64679-0

**Published:** 2024-06-18

**Authors:** Jia Kang, Bingcheng Wan, Zhiqiu Gao, Shaohui Zhou, Huansang Chen, Huan Shen

**Affiliations:** School of Atmospheric Physics, Nanjing University of Information Science and Technology, Nanjing, 210044 China

**Keywords:** Natural hazards, Mathematics and computing

## Abstract

Landslides are highly destructive geological disasters that pose a serious threat to the safety of people’s lives and property. In this study, historical records of landslides in Yunnan Province, along with eight underlying factors of landslide (elevation, slope, aspect, lithology, land cover type, normalized difference vegetation index (NDVI), soil type, and average annual precipitation (AAP)), as well as historical rainfall and current rainfall data were utilized. Firstly, we analyzed the sensitivity of each underlying factor in the study area using the frequency ratio (FR) method and obtained a landslide susceptibility map (LSM). Then, we constructed a regional rainfall-induced landslides (RIL) probability forecasting model based on machine learning (ML) algorithms and divided warning levels. In order to construct a better RIL prediction model and explore the effects of different ML algorithms and input values of the underlying factor on the model, we compared five ML classification algorithms: extreme gradient boosting (XGBoost), k-nearest neighbor (KNN), support vector machine (SVM), logistic regression (LR), and random forest (RF) algorithms and three representatives of the input values of the underlying factors. The results show that among the obtained forecasting models, the LSM-based RF model performs the best, with an accuracy (ACC) of 0.906, an area under the curve (AUC) of 0.954, a probability of detection (POD) of 0.96 in the test set, and a prediction accuracy of 0.8 in the validation set. Therefore, we recommend using RF-LSM model as the RIL forecasting model for Yunnan Province and dividing warning levels.

## Introduction

Landslide involves the downward movement of rocks, soil, and organic matter along slopes under the influence of gravity. It is characterized by their large number, wide extent, intense activity, and suddenness. Also it is the main type of geologic disaster that threatens the safety of human life, causes economic and property losses, and restricts the development of social economy and civilization^1^. China is one of the countries most severely affected by geological disasters, with landslides accounting for a significant proportion. Among the main triggers of landslides, rainfall is a highly active and variable natural factor, with approximately 90% of landslides being triggered by rainfall^2–4^.

Currently, the prediction of RIL can be categorized into two main types: critical rainfall models and probabilistic forecasting models. (1) Critical rainfall models directly link landslide occurrence to rainfall by identifying empirical relationships between various rainfall indicators and landslide occurrences to determine critical rainfall conditions^5,6^. There are various empirical methods available for spatiotemporal prediction of RIL , which have been applied in disaster assessment or warning systems worldwide^7^. The most common approach involves considering both rainfall intensity and duration thresholds^8^. Earlier, Aleotti^9^ analyzed four typical cases to determine the empirical rainfall thresholds in the Pearson area and derived different empirical formulas for the thresholds. Later, Bui et al.^10^, Jaiswal and Westen^11^, obtained different forecast and warning models by establishing different thresholds in different study areas according to different geological conditions. Recently, Huang et al.^12^ constructed a new threshold warning model by linking LSMs and thresholds for forecasting. (2) Probabilistic forecasting models predict the probability of geological hazard occurrence under different underlying surface conditions using rigorous statistical methods. In the past few years, the construction of regional RIL warnings through mathematical-statistical models has received a lot of scholarly attention, which allow for dynamic forecasting of landslides with spatial-temporal probabilities^7,13,14^. Similarly, ML algorithms have been proposed for dynamic prediction of landslides in some cases. For instance, Dai et al.^15^ developed a logistic regression model that preliminarily possesses the dynamic forecasting capability for RIL probabilities in the Dayu Mountain area. Dynamic predictive assessment of global landslide risk by the landslide hazard assessment for situational awareness (LHASA) model using XGBoost^16^. Due to its unique geographical and climatic conditions, landslide disasters in Yunnan Province, China, are particularly pronounced. Traditional empirical threshold landslide prediction systems still face uncertainties in forecasting in this region^17^. The application of ML algorithm techniques has greatly enriched the prediction and early warning methods for geologic hazards such as landslides and mudslides. These algorithms have been widely used in recent years due to their ability to process multidimensional data and synthesize spatial information^18^. In order to explore the differences between different ML for constructing RIL forecasting models and the influence of the input values of the underlying factors on the models. Building upon previous research, this study combines eight underlying factors affecting landslides (elevation, slope, aspect, lithology, land cover type, NDVI, soil type, and AAP) and historical rainfall data with daily rainfall data to compare five ML methods (XGBoost, SVM, KNN, LR, RF). Meanwhile, we explore the effect of the FR method to deal with the underlying factor on the ML model, and try to construct an optimal RIL forecasting and warning model for Yunnan Province.

“Materials and methods” section discusses data and methods, including data sources and processing, model building process methods and evaluation indicators, “Results and discussion” section discusses the analysis and application of results, and “Conclusion and outlook” section summarizes and outlooks the article.

## Materials and methods

### Study area and datasets

Yunnan Province is located in the southwest of China, with coordinates ranging from $21^\circ 8'$N to $29^\circ 15'$N and $97^\circ 3'$E to $106^\circ 11'$E. It has an average elevation of around 2000 m^19^. The terrain of Yunnan Province exhibits a trend of higher elevation in the northwest and lower elevation in the southeast, with significant variations in elevation. The geological evolution of Yunnan Province has been influenced by multiple tectonic movements, including the Yanshan Movement and neotectonic movements, as well as intense fault activity, significant differential uplift, and strong river incision processes. Among the various landforms in Yunnan Province, slopes are the most predominant and occupy the largest percentage of the landforms. Approximately 86% of the province’s area is characterized by mountainous terrain, with a small portion comprising basin areas, mostly distributed within the mountainous regions^20^.

**Figure 1:**
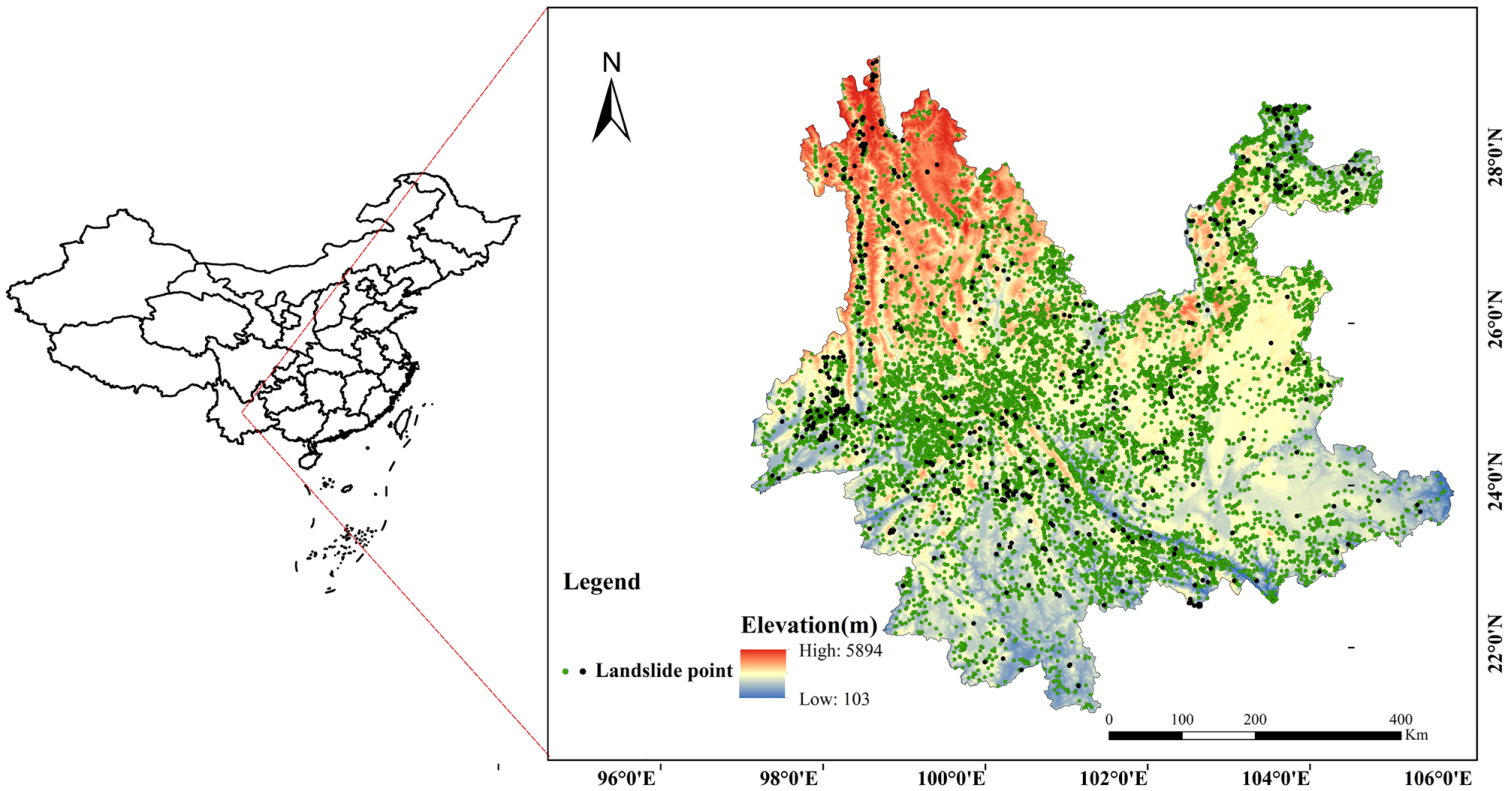
Distribution of study area and landslides (green dots represent the distribution of all landslides , black dots represent landslides labeled with date).

The landslide dataset used in this study is sourced from the Geographic Remote Sensing Ecological Network Scientific Data Registration and Publishing System. It consists of two datasets, one with location information of 11,610 landslides in Yunnan Province, and the other with a number of landslide records containing the date of landslide occurrence. Due to incomplete date records in the dataset, 256 landslide cases from year of 2016 were selected for model training and testing, with an additional 20 cases from 2014 used as a validation set. Figure 1 illustrates the geographical location and elevation distribution of Yunnan Province, along with the spatial distribution of landslide disaster points.

**Figure 2:**
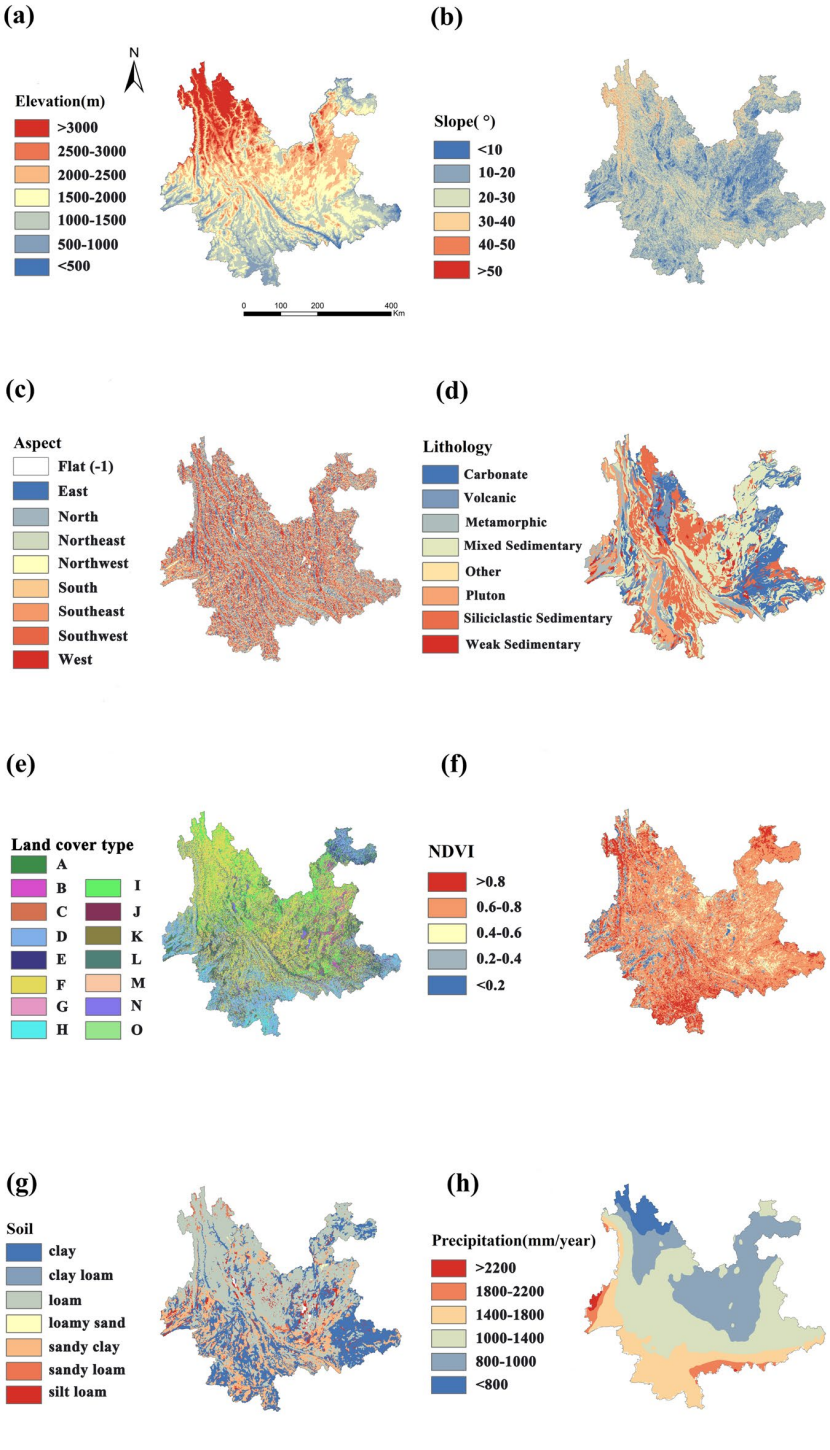
Spatial distribution of subsurface factors after categorization (**a** elevation, **b** slope, **c** aspect, **d** lithology, **e** land cover type, **f** NDVI, **g** soil, **h** AAP).

The elevation data were obtained from ASTERG_DEM 30 m $\times$ 30 m resolution data, and slope and aspect were computed using ArcGIS 10.8 software. Lithology data were sourced from the 1 km $\times$ 1 km lithology data of the LHASA 2.0 version. Land cover type data utilized 30 m $\times$ 30 m resolution nationwide vegetation cover type data. NDVI data were derived from the MOD13A3 dataset regularly released by NASA and were generated by the maximum synthesis method. Soil type data were sourced from the 1km horizontal resolution soil database launched by the Food and Agriculture Organization (FAO) of the United Nations. AAP was calculated from the daily rainfall data of the GPM project from 2001 to 2020. All underlying surface factors were standardized to a 1 km $\times$ 1 km resolution. The sources of each factor’s data are listed in Table 1, and their spatial distribution is illustrated in Fig. 2.

**Table 1:** Underlying factors and data sources.

Underlying factors	Data source
Elevation	https://www.gscloud.cn/
Slope	
Aspect	
Lithology	https://github.com/nasa/LHASA
Average annual precipitation	https://disc.gsfc.nasa.gov/datasets/
Land cover type	http://www.globallandcover.com/
Normalized difference vegetation index (NDVI)	https://search.earthdata.nasa.gov/search
Soil	https://www.fao.org/

### **Processing of characterization factors**

#### Rainfall factors

The process of RIL is typically categorized into two types: short-duration heavy rainfall and prolonged rainfall. Therefore, when considering rainfall-induced landslides, it is essential to not only assess the precipitation on the day of occurrence but also take into account preceding rainfall events . Effective rainfall refers to the portion of rainfall that has an inducing effect on landslide occurrence. This concept was initially proposed by Crozier in 1980^21^ and has since been applied in the study of rainfall thresholds for geological hazards. In recent years, it has been widely utilized in research on RIL^7^. The formula for calculating effective rainfall is as follows:

1 $$\begin{aligned} P_a = R_0 + KR_1 + K^2R_2 + K^3R_3 + \ldots + K^nR_n \end{aligned}$$

$P_a$ represents the effective rainfall, *K* is the rainfall attenuation coefficient, $R_0$ is the rainfall on the day of the landslide, $R_1$ is the rainfall on the next day, and so on, and *n* represents the number of days before the landslide.

To compute effective rainfall, it is necessary to determine the parameters *n* and *K*. This study conducted a statistical analysis of 256 landslide samples, examining the daily cumulative precipitation for the 15 days preceding landslide occurrences, as well as the frequency of occurrence for different levels of precipitation. The analysis revealed that there is a large value center of landslide disasters around 11 days, with disasters ranging from 75 to 90, corresponding to a cumulative sliding precipitation of about 100 mm, and no disaster samples are in a situation where the cumulative precipitation is less than 25 mm. Before this large value center, there are some disaster samples during periods when the cumulative precipitation is less than 25 mm, which is inconsistent with the actual situation^17^. Consequently, this study uniformly selected a preceding cumulative rainfall period of 11 days (*n* = 11). Additionally, a *K* value of 0.84 was adopted as the rainfall attenuation coefficient. Although this parameter was derived from data in a specific region of North America, validation results across various global regions have demonstrated the applicability and effectiveness of this method and parameter setting^22^.

The conventional method for acquiring rainfall information at landslide sites involves estimating rainfall records from nearby ground observation stations. However, due to factors such as terrain and economic considerations, the ground observation station network is often sparsely and unevenly distributed, leading to inaccuracies in the spatial and temporal distribution of regional rainfall. Consequently, this affects the research on forecasting and early warning of rainfall-induced landslides. In order to obtain more accurate rainfall data at landslide sites, this study utilizes daily precipitation data from the Global Precipitation Measurement (GPM) project for spatial interpolation analysis to derive rainfall data at landslide points. Subsequently, effective rainfall analysis is conducted.

#### Underlying factors

The occurrence of RIL requires the combined influence of underlying surface conditions and precipitation. Since the composition of underlying surface environments is complex and different factors play varying roles, considering the contribution of various factors to landslide occurrence is crucial. The FR model enables the quantitative analysis of the relationship between landslide points and the various factors influencing landslides^23^. By calculating the proportion of landslides occurring in different classification intervals of various underlying factors to the proportion of the area influenced by these factors, the FR for all influencing factors is summed to obtain the landslide susceptibility index. The formula for calculating the FR is as follows:

2 $$\begin{aligned} FR = \frac{\frac{N_i}{N}}{\frac{S_i}{S}} \end{aligned}$$

$N_i$ is the area of landslides occurring in a subset or interval of a given factor; N is the total area of landslides in the study area; $S_i$ is the area of a sub-interval or subset of a given indicator; S is the total area of the study area. When *FR* > 1 of the influence factor, it represents that the evaluation factor contributes to landslides; *FR* = 1, represents that the evaluation factor does not have a significant effect on landslides; $FR<$ 1, indicates that the evaluation factor has little relevance to landslides^24^. In this study, the widely recognized underlying factors were selected, and elevation, slope, aspect, lithology, land cover type, NDVI, soil type, and annual precipitation were chosen as the input factors to the FR model, and the FR value of each underlying factor were calculated by combining the entire landslide record of 11,610 landslide individual cases^25^. The results of the FR value calculations for various underlying surface factors are presented in Table 2.

**Table 2:** Results of the calculation of the frequency ratio of the underlying factors.

Elevation (m)	Rasters	Disasters	FR	NDVI	Rasters	Disasters	FR
< 500	2111	106	1.66	< 0.2	13,679	247	0.60
500–1000	29,237	923	1.04	0.2–0.4	20,500	410	0.66
1000–1500	88,462	2815	1.05	0.4–0.6	59,933	1687	0.93
1500–2000	121,056	4914	1.34	0.6–0.8	225,608	8077	1.18
2000–2500	81,744	2328	0.94	>0.8	63,062	1172	0.61
2500–3000	28,891	414	0.47				
> 3000	31,729	103	0.11				
Slope (^∘^)	Rasters	Disasters	FR	Lithology	Rasters	Disasters	FR
< 10	41,553	942	0.75	Other	72	2	0.92
10–20	157,434	5092	1.07	Weak sedimentary	11,894	224	0.62
20–30	134,836	4313	1.06	Volcanic	24,111	562	0.77
30–40	42,983	1143	0.88	Siliciclastic sedimentary	90,066	2523	0.93
40–50	5909	111	0.62	Mixed sedimentary	140,219	5223	1.23
> 50	354	3	0.28	Metamorphic	23,535	983	1.38
				Carbonate	66,696	1532	0.76
				Pluton	26,582	550	0.68
Aspect (^∘^)	Rasters	Disasters	FR	Average annual precipitation (mm/year)	Rasters	Disasters	FR
Flat (–1)	536	0	0.00	<800	15,556	167	0.35
North (337.5–22.5)	38,681	1271	1.08	800–1000	119,091	3842	1.06
Northeast (22.5–67.5)	46,992	1535	1.08	1000–1400	154,433	5395	1.15
East (67.5–112.5)	55,605	1739	1.03	1400–1800	77,242	1819	0.77
Southeast (112.5–157.5)	49,749	1461	0.97	1800–2200	11,930	316	0.87
South (157.5–202.5)	40,771	1120	0.90	>2200	1732	25	0.47
Southwest (202.5–247.5)	46,618	1292	0.91				
West (247.5–292.5)	54,126	1635	0.99				
Land cover type	Rasters	Disasters	FR	Soil	Rasters	Disasters	FR
Dryland (A)	30,235	1781	1.95	Clay	114,186	3322	0.96
Meadow (B)	18,485	650	1.16	Clay loam	134	8	1.97
Paddy field (C)	1945	44	0.75	Silt loam	16,888	730	1.42
Evergreen broadleaf forest (D)	57,453	1111	0.64	Loam	179,965	5232	0.96
Deciduous broadleaf forest (E)	40,261	1581	1.30	Sandy clay loam	67,053	2191	1.08
Evergreen needleleaf forest (F)	134,999	3236	0.79	Sandy loam	3043	83	0.90
Shrubland (G)	13,276	498	1.24	Loamy sand	631	26	1.36
Bush woodland (H)	20,114	461	0.76				
Grassland (I)	56,748	1905	1.11				
Sparse woodland (J)	1	0	0.00				
Wetland (K)	11	0	0.00				
Urban area (L)	6238	280	1.48				
Bare area (M)	115	1	0.29				
Water body (N)	2818	46	0.54				
Permanent glacier (O)	249	0	0.00				

**Figure 3:**
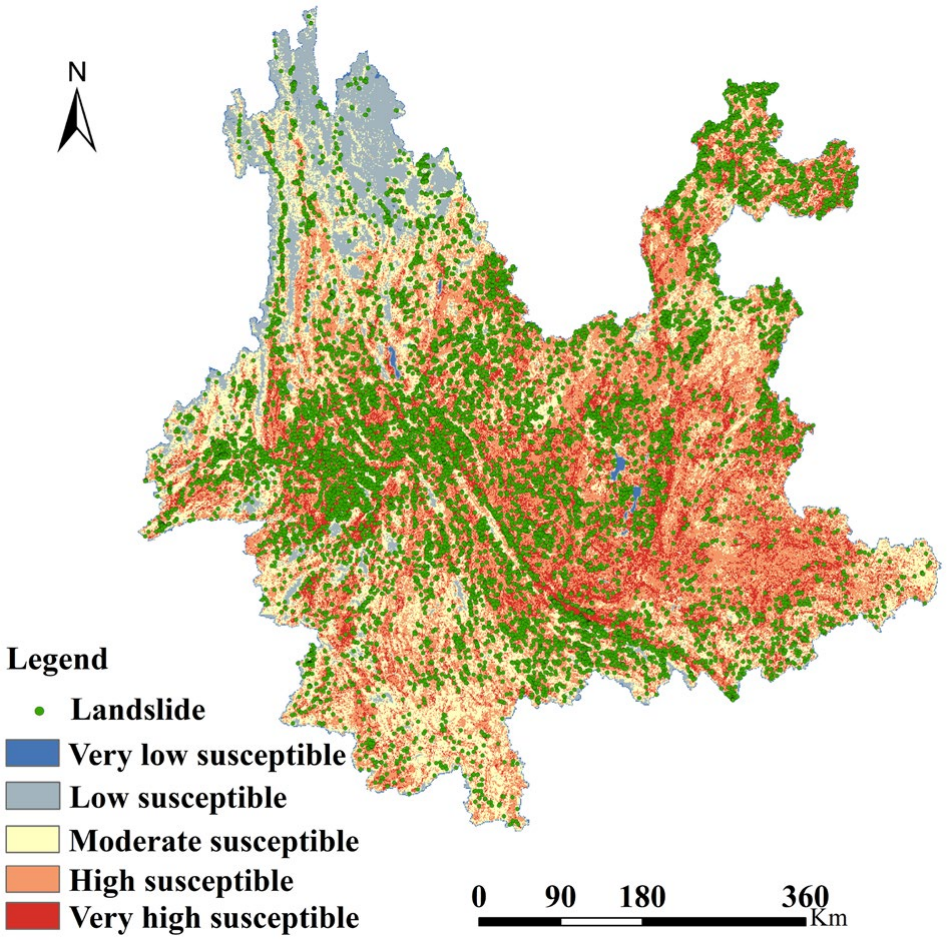
Landslide susceptibility map.

We map the FR values calculated in Table 2 to the eight underlying factors in Fig. 1, and then overlay the eight layers to obtain a comprehensive susceptibility index map. Then we use the natural breakpoint method in Arcgis10.8 to divide it into five levels^26^. Consequently, we get the landslide susceptibility map for Yunnan Province (Fig. 3). From Fig. 3, it is evident that there are several areas in Yunnan Province classified as landslide-prone zones. The largest susceptible zone is predominantly situated in the central and eastern parts of Yunnan Province, while the susceptibility to landslides in the northwestern and southern regions is notably lower compared to other areas.

### **Model evaluation metrics**

In comparing model superiority, commonly used indicators include ACC, POD, probability of false detection (POFD), and AUC. ACC represents the proportion of correctly classified samples in the test set relative to the total sample size, evaluating the predictive accuracy of the model. The receiver operating characteristic curve (ROC) is a comprehensive indicator of the sensitivity and specificity of a continuous variable, and AUC evaluates the generalization ability of the model^1^. A higher AUC value, closer to 1, indicates better differentiation capability between different categories; when AUC approaches 0, the model’s discriminative ability is poor, potentially leading to erroneous predictions, and at 0.5, the model cannot differentiate between categories. AUC is considered one of the most useful metrics in landslide model evaluations^27^. The POD indicates the probability that the algorithm correctly detects an actual occurrence; the POFD indicates the probability that an actual occurrence does not occur, but the algorithm forecasts it occur. They are scored between 0 and 1 and are calculated as follows:

3 $$\begin{aligned} ACC= & {} \frac{TP + TN}{TP + TN + FP + FN + TP} \end{aligned}$$

4 $$\begin{aligned} POD= & {} \frac{TP}{TP + FN} \end{aligned}$$

5 $$\begin{aligned} POFD= & {} \frac{FP}{FP + TN} \end{aligned}$$

Where *TN* denotes true negatives, indicating actual occurrences of false being predicted as false; *TP* represents true positives, indicating actual occurrences of true being predicted as true; *FP* refers to false positives, signifying actual occurrences of false being predicted as true; and *FN* represents false negatives, indicating actual occurrences of true being predicted as false.

### **Model built**

**Figure 4:**
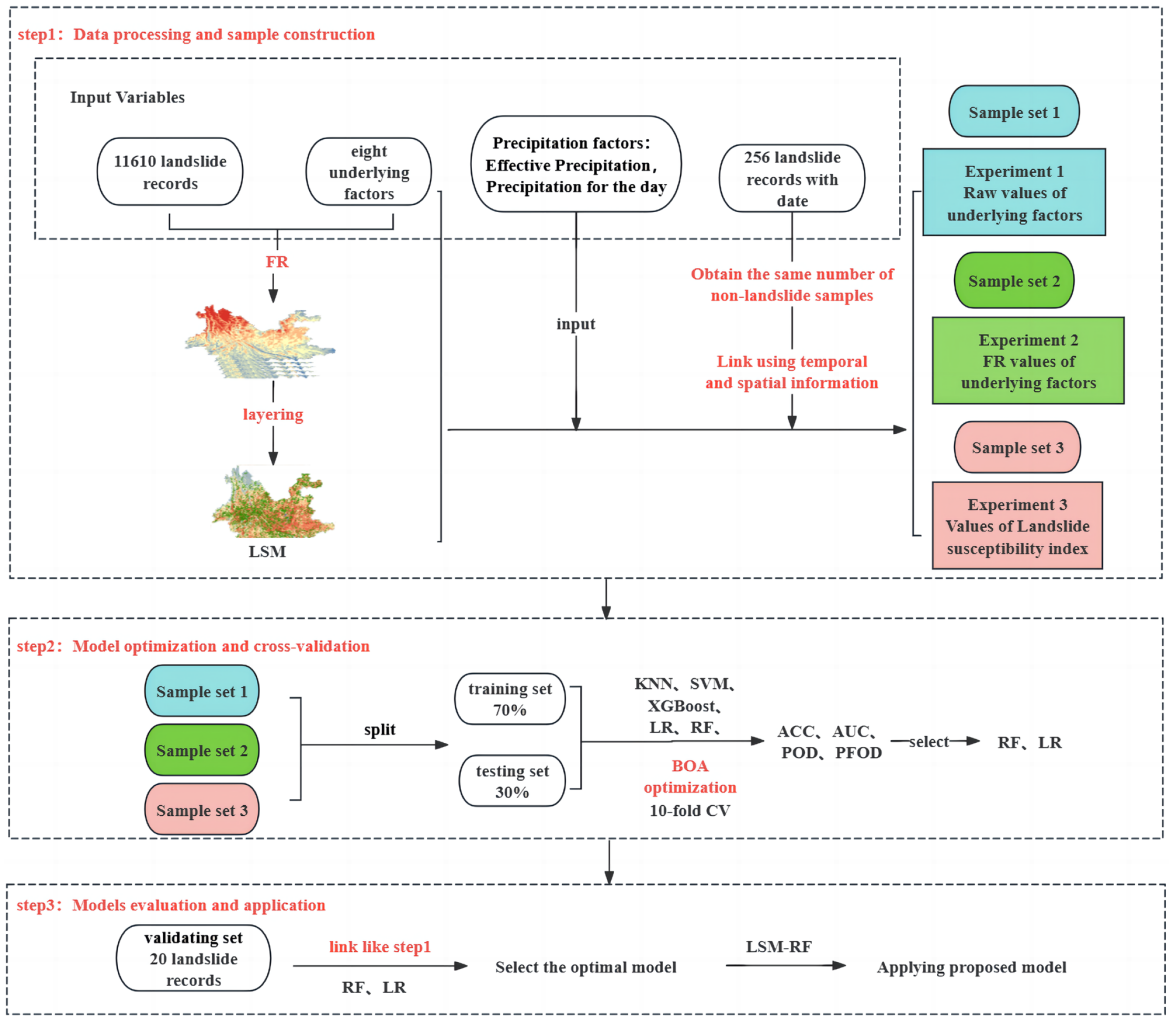
Flowchart of model construction.

The construction of the RIL forecasting model in this study is mainly divided into three steps, and five machine learning algorithms are used in the process, three sample sets are constructed according to the different treatments of the underlying factors, and a total of 15 experiments are conducted. The main flow of the experiments is shown in Fig. 4.

The first step involves data processing and three sample sets construction. The construction process of the sample sets mainly includes the establishment of underlying factors and rainfall factors databases, selection of positive and negative samples, extraction of sample feature attributes, and quality control. In this study, 256 landslide cases recorded in 2016 are used as positive samples. An equal number of non-landslide data are randomly selected in terms of time and space to form complete landslide samples. Subsequently, feature attributes of the corresponding underlying factors and rainfall factors are extracted based on the time and spatial information of the landslide samples. Due to the complexity of the underlying environment and to investigate the impact of underlying factors processed through FR methods on machine learning models, three sample sets are constructed for comparative analysis. The first sample set directly extracts the original data values of each underlying factor as feature values. The second sample set replaces the original values with the FR values calculated for each underlying factor as feature values. The third sample set uses the susceptibility index obtained by overlaying the FR values of the eight underlying factors as feature values. Additionally, the effective rainfall and daily precipitation for the corresponding time are extracted as features to form complete three sample sets. These are respectively referred to as Sample 1, Sample 2, and Sample 3 in this study.

The second step involves model optimization and cross-validation. In this study, the three sample sets from the first step are split into training and testing sets in a 7:3 ratio and evaluated using tenfold cross-validation. For each of these three sample sets, five machine learning algorithms (XGBoost, SVM, KNN, LR, RF) are employed to construct different machine learning models. Adjusting the input parameters of the models can significantly improve the effectiveness and stability of the models. Therefore, Bayesian optimization algorithm (BOA) is used in this study to fine the parameters of all models, thereby obtaining the optimal hyperparameters for each model^28^.

The third step involves model evaluation and application. This part primarily assesses the superiority of the models using classification algorithm evaluation metrics such as ACC, AUC, POD, and POFD. After selecting more accurate and stable algorithms, validation and application are carried out using 20 landslide cases recorded in 2014. This process aims to obtain the optimal rainfall-induced landslide prediction model for Yunnan Province and subsequently establish model warning levels. When applying this landslide prediction and warning model, it is essential to input the underlying factors layers, effective rainfall layers, and forecast rainfall layers to obtain the prediction and warning results for landslides.

## Results and discussion

### **Machine learning algorithm analysis**

The evaluation metrics scores of five ML algorithms are obtained through the first step and second step of the above model building part, as shown in Table 3.

The results from Sample Set 1 reveal significant differences among various machine learning models in rainfall-induced landslide prediction. Among them, XGBoost, LR, and RF show better performance, while SVM performs the worst, with an AUC of only 0.537.

After applying the FR method to the underlying surface factors in Sample Set 2, we find that the ACC of all five ML algorithms improved. The SVM showed the most significant improvement, while the RF showed the least improvement. In addition, all the ML models have an ACC greater than 0.9, with the LR algorithm having the highest ACC; considering the accurate prediction of disasters, on POD, XGBoost, LR, and RF still perform better.

In Sample Set 3, after representing the susceptibility index as a representative feature for the eight underlying factors, the computational efficiency improves as the number of features for ML decreases from eight to one. However, there is a slight decrease in the ACC of the five ML models. Their ACC values are still all around 0.9. Among them, RF has the highest ACC of 0.906, with an AUC of 0.954 and a POD of 0.96, but it also exhibits a relatively high false alarm rate, with a POFD of 0.15. Comparing the performance of the same ML method across the three sample sets, we observe that Sample Set 2 and Sample Set 3 have higher ACC values compared to Sample Set 1. This suggests that the FR method for preprocessing underlying factor data contributes to the application of ML in RIL prediction. Additionally, comparing the model performance between Sample Set 2 and Sample Set 3, we find that Sample Set 2 performs slightly better, but the difference between the two is so small. This indicates that the landslide susceptibility index represents the underlying surface environment adequately, highlighting the importance of accurate susceptibility map research.

Among the five machine learning methods, RF and LR consistently perform well across all sample sets, with ACC, AUC, and POD values all above 0.9. RF exhibits the most stable performance. In conclusion, LR and RF are more stable and accurate compared to other machine learning models, making them more suitable as RIL prediction models.

**Table 3:** Performance metrics for different models on different sample sets.

Model	Sample Set 1				Sample Set 2				Sample Set 3			
	ACC	AUC	POD	POFD	ACC	AUC	POD	POFD	ACC	AUC	POD	POFD
KNN	0.698	0.754	0.600	0.200	0.916	0.975	0.900	0.070	0.899	0.939	0.960	0.160
XGBoost	0.903	0.964	0.940	0.090	0.913	0.966	0.940	0.090	0.893	0.941	0.920	0.140
SVM	0.512	0.537	0.060	0.000	0.916	0.945	0.900	0.070	0.886	0.887	0.940	0.160
LR	0.900	0.958	0.910	0.110	0.936	0.971	0.950	0.080	0.900	0.950	0.910	0.110
RF	0.903	0.957	0.900	0.090	0.910	0.964	0.930	0.110	0.906	0.954	0.960	0.150

### **Case discussion**

#### Determination of the model

Based on the algorithm evaluation results, LR and RF are ultimately selected for practical application. 20 landslide cases recorded in 2014, unrelated to the previous sample sets, are selected. Using the spatiotemporal distribution information of landslide points, rainfall factor information, and underlying factors information, the prediction accuracy was calculated to validate the model, and the optimal RIL prediction model is chosen.

**Figure 5:**
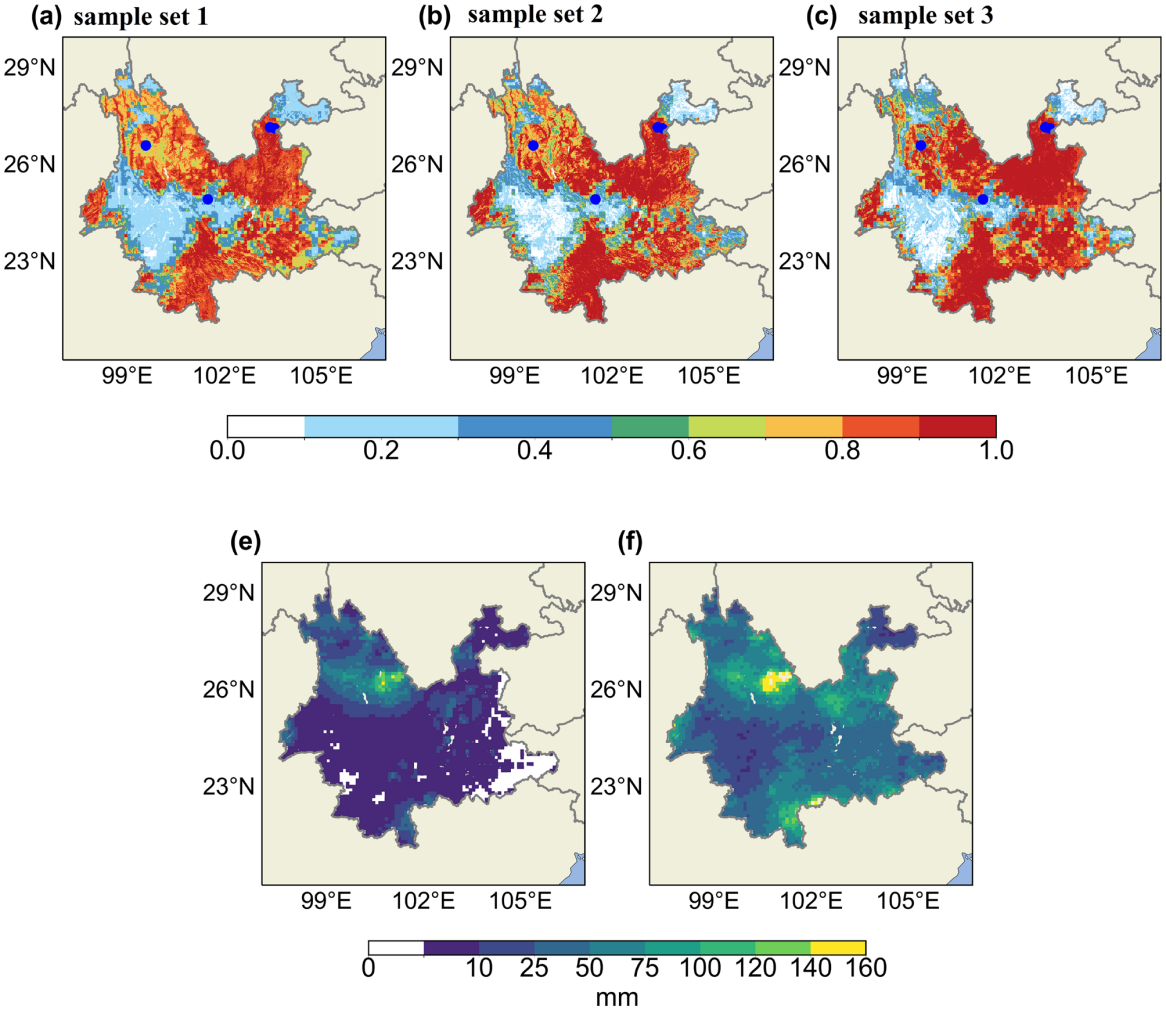
(**a**–**c**) Predictions for 2014-07-06 from three different sample (the blue dots represent the recorded landslides that occurred) (**e**) rainfall for the day, (**f**) effective rainfall.

The prediction accuracy rate of LR algorithm for the 2014 dataset samples are 0.65, 0.70, and 0.75 respectively for the three sample sets, while for RF algorithm, the prediction accuracy rates are 0.80, 0.75, and 0.80. The accuracy of RF algorithm is higher than LR across all three sample sets. Therefore, the RF algorithm is ultimately chosen to establish the RIL prediction and warning model for Yunnan Province. For a more intuitive understanding of the spatial forecasting performance of the three sample sets using the RF model, we selected the forecast results for July 6, 2014 (Fig. 5). From the graph, it can be observed that the forecasted landslide extents are generally similar across the three sample sets,with high prediction accuracy. This indicates that our prediction line for RIL using RF and FR is stable and reliable. However, there is a considerable false alarm area in the forecast. Based on the rainfall information (Fig. 5e. Rainfall on the day, f. Effective rainfall), it can also be seen that the probability distribution of landslide occurrence has a close relationship with the distribution of rainfall, and in the area of the false alarm of the landslide forecast, the effective rainfall is higher even though the rainfall is very small on the day, which indicates that the area of the false alarm is not a certainty. It’s essential to consider that the landslide record dataset represents only a subset of actual landslide occurrences. Additionally, in real-world geological disaster forecasting and warning operations, missing warnings are far more serious than false alarms. Therefore, ensuring high hit rates and low miss rates during the training process of classification algorithm models is a more critical prerequisite. This study focused on the selection of RIL samples more, leading to the misclassification of some non-landslide samples as landslide samples. Consequently, there is a considerable extent of false alarms in the actual forecasting and warning. In the future, it is necessary to increase the inclusion of negative samples containing rainfall processes while maintaining a high hit rate, to improve the classification algorithm model’s ability to identify non-landslide rainfall processes and reduce the false alarm rate^5^.

Comparing the forecasting results of the three sample sets, it can be seen that the RF model of sample set 3 shows more concentrated predictions in both high and low probabilities. This is advantageous for disaster forecasting; however, it also leads to an increase in false alarms and misreports while increasing the hit rate. This is reflected in the POD = 0.96 and POFD = 0.16 of sample set 3, RF, as shown in Table 3. Considering that the more input features in ML, the longer the computation time, the RF model based on landslide susceptibility maps is ultimately chosen as the optimal model for the study area.

#### Classification of early warning levels

**Figure 6:**
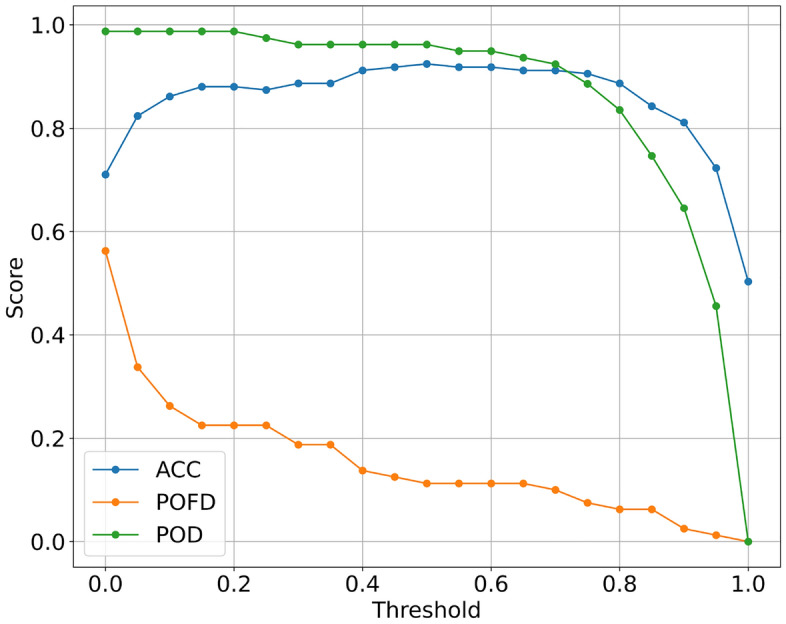
Changes in evaluation metrics with model thresholds.

When the results of the forecast output need to be applied in the actual , we needs to be divided into warning levels according to the probability value, in order to better warn and implement the work. To establish more precise forecasting and warning divisions, we adjust the model’s decision threshold. We do this by observing how ACC, POD, and POFD change with the model’s threshold (as shown in Fig. 6) to reasonably define the warning levels. In Fig. 6, it can be observed that before 0.2, ACC continues to improve, the POFD decreases rapidly. Between 0.2 and 0.55, there is a stable ACC and POD. Between 0.55 and 0.7, the POFD continues to decrease. After 0.7, both ACC and POD exhibit rapid declines. Therefore, this study sets the following warning level divisions: 0–0.2: no warning, 0.2–0.55: low-level warning, 0.55–0.7: moderate-level warning, and 0.7–1.0: high-level warning. After dividing the warning levels, 90% of them were in warning status, and 75% of them were high level warnings, 5% intermediate level warnings, and there were only 2 cases of landslides in no warnings, which reflects the model’s ability to warn of typical RIL.

## Conclusion and outlook

In this study, a forecasting and warning model for RIL in Yunnan Province based on susceptibility maps and RF is finally obtained, and the following conclusions are obtained in the process:

- Pre-processing of underlying factors by FR methods can improve ML scores in RIL prediction Moreover, susceptibility maps derived from the FR exhibit a certain level of representativeness for the underlying environment, highlighting the necessity of LSM research.
- Among the various machine learning models including KNN, XGBoost, SVM, LR, and RF used in the RIL forecasting model, the RF and LR models exhibit the best performance. They achieve an ACC, AUC, and POD all exceeding 0.9, surpassing the performance of other machine learning models on test set. Specifically, the RF model demonstrates the most stable performance. Furthermore, on the validation set, RF outperforms LR.
- The LSM-RF model yields the optimal RIL forecasting model for the study area. The RF models of the three sample sets perform similarly in spatial extent forecasting, with accurate forecasts but larger false alarm areas; among them, the RF model based on the susceptibility map is more concentrated in the high and low probability forecasts; the model has a prediction accuracy of 0.8 in the test set ACC = 0.906, AUC = 0.954, and POD = 0.96, and the validation set prediction accuracy is 0.8.
- We propose to divide the model warning levels according to the change of evaluation metrics with model thresholds. Finally, the warning levels for the research area are defined as follows: no warning for probabilities between 0 and 0.2, low-level warning for probabilities between 0.2 and 0.55, moderate-level warning for probabilities between 0.55 and 0.7, and high-level warning for probabilities between 0.7 and 1. Thus, the RIL forecasting and warning model for Yunnan Province is established.

Landslide prediction is a challenging task due to the complex interactions among multiple triggering factors. Moreover, the inherent uncertainty in the data used to develop landslide models adds further complexity to the research. The lack of specific dates for many landslides in the dataset poses a significant challenge to our study. However, compared to traditional threshold-based empirical models, ML-based prediction models have made significant progress in quantifying the probability of landslide occurrence^17,29,30^.

In the future, we plan to enhance the predictive capabilities of our model by refining and augmenting the landslide dataset and leveraging the timeliness of satellite data. Additionally, employing alternative mathematical methods to derive more accurate susceptibility maps will further improve the predictive performance of the model. As for the selection of the value of K, the attenuation coefficient of the previous rainfall, some articles have different opinions^31^ , and it is expected that more detailed values can be obtained in the future to further optimize the model. Furthermore, integrating the Weather Research and Forecasting (WRF) model for fine-scale precipitation forecasting, along with variables related to soil moisture, may enhance the effectiveness of regional RIL prediction and warning systems.

## Data Availability

Some of the data sources are shown in Table 1 of the manuscript, and other data that support the findings of this study have been deposited in this website: https://github.com/j-kang666/landslides. The maps in this manuscript are derived from articles^32^ by Z.S, who is also one of the authors of this manuscript. We used arcgis10.8:https://desktop.arcgis.com/zh-cn/desktop/index.html and python3.8: https://www.python.org/ to plot Figs. 1, 2, 3, and 5 in the manuscript.
